# Bilateral Open Globe Injury Secondary to the King of Fruits: Durian Fall

**DOI:** 10.7759/cureus.39153

**Published:** 2023-05-17

**Authors:** Jing Lee, Jemaima Che Hamzah, Safinaz Mohd Khialdin, Ainal Adlin Naffi

**Affiliations:** 1 Department of Ophthalmology, Faculty of Medicine, Universiti Kebangsaan Malaysia, Kuala Lumpur, MYS

**Keywords:** personal protective equipment, wound repair, ocular emergency, open globe injury, durian

## Abstract

We report a case of bilateral open globe injury that resulted from a durian fruit falling on a 62-year-old woman's unprotected face during durian picking in her orchard. On presentation, the bilateral vision was light perception. The right eye sustained a curvilinear corneal laceration with expelled intraocular content. Meanwhile, the left eye sustained a corneoscleral laceration with expelled uvea and retina. Additionally, the right upper lid margin was lacerated. Emergency wound exploration, primary toilet, and suturing were performed on bilateral eyes. Preoperatively, she received intramuscular anti-tetanus toxoid and intravenous ciprofloxacin. Intravitreal ceftazidime and vancomycin were given intraoperatively as endophthalmitis prophylaxis. Postoperatively, the vision remained as light perception. There were no signs of endophthalmitis in both eyes. Although traumatic globe injury due to durian is uncommon, individuals should wear protective gear while in a durian orchard to avoid such unprecedented accidents. Prompt yet scrupulous action should be taken to save the globe and further possible complications.

## Introduction

Durian (*Durio zibethinus*) is a seasonal fruit that is popular in Southeast Asia region despite its pungent smell. Named in some regions as the "king of fruits," durian is distinctive for its thorn-covered rind and large oblong or round size. The size of durians can reach up to 30 cm in length, 15 cm in diameter, and eight kg in weight [1]. Durian trees can grow up to 40 meters in height in tropical forests [2]. During durian season, people wait for the ripe durian to fall and collect it from the orchard. Nevertheless, safety precautions and awareness while in the orchard are still lacking, especially in rural areas. Aziz et al. and Reddy reported a total of four cases regarding unilateral open globe injuries (OGIs) secondary durian fall [3,4]. The latest incident was reported in 2021 by Ibramsah et al. [5].

This article was previously presented as a poster at the 14th Asia-Pacific Vitreo-retina Society (APVRS) Congress on December 11-12, 2021.

## Case presentation

A 62-year-old lady was brought to the emergency department (ED) for loss of consciousness and bilateral loss of vision after a durian fell on her face while she was picking durians in her orchard. The mechanism of injury was unclear; however, she did not wear any protective gear during the incident.

**Figure 1:**
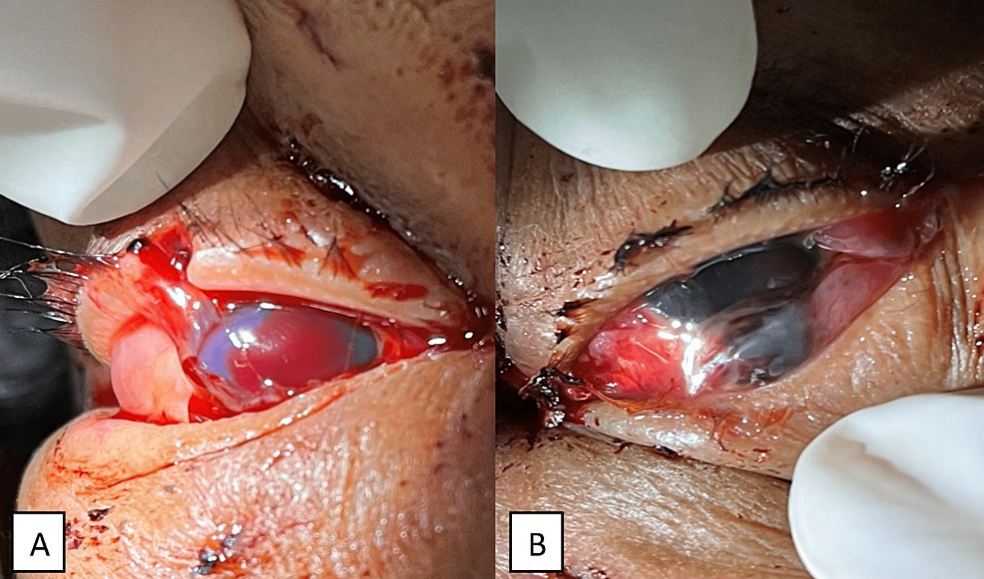
(A) Right upper lid and corneal laceration with total hyphema and expelled intraocular content. (B) Left corneoscleral laceration with expelled uveal and retinal tissue.

She sustained multiple puncture wounds on her face with soft tissue swelling and a right upper lid laceration. Both eyes' visual acuity was light perception (LP). Relative afferent pupillary defect (RAPD) was not possible to assess. Right eye examination showed a curvilinear corneal laceration with expelled intraocular content (Figure 1A). However, further findings were limited by total hyphema. Meanwhile, her left eye sustained a corneoscleral laceration inferiorly measuring about 11 mm with expelled uveal and retinal tissue (Figure 1B). She was given intramuscular anti-tetanus toxoid 0.5 ml and intravenous ciprofloxacin 500 mg stat in the ED.

**Figure 2:**
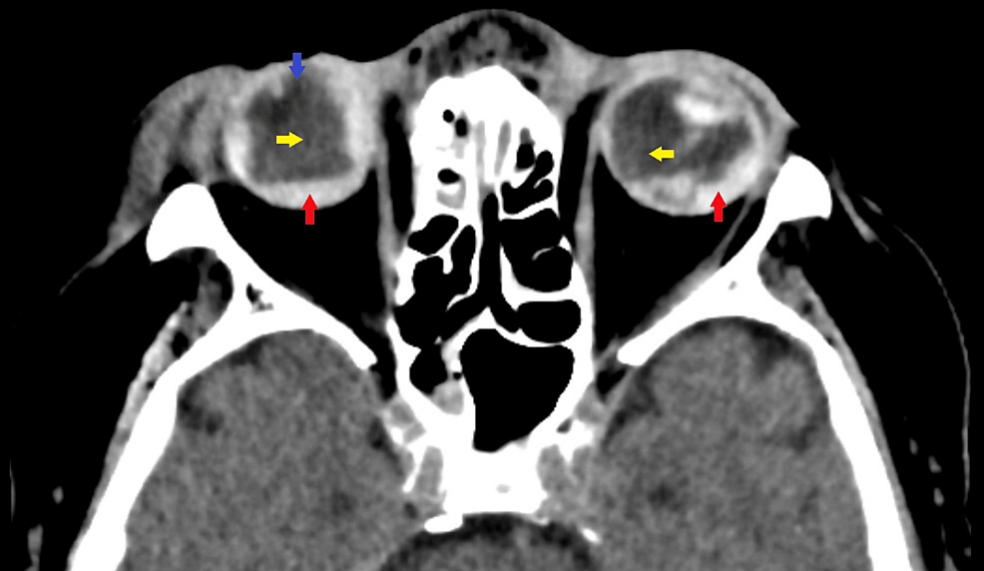
Axial cut CT of the brain and orbit showed bilateral globes were deformed, which was suggestive of globe rupture. Subretinal hyperintensities were seen in both orbits suggestive of hemorrhagic retinal detachment (red arrow). Vitreous hyperintensities indicated vitreous hemorrhage (yellow arrow). The absence of the right crystalline lens was also seen (purple arrow).

After she was medically stabilized, an emergency exploration of the wound was carried out under general anesthesia in the operating theater to look for the extent of the injury and primary repair (Figure 2).

Intraoperative findings of the right eye showed a mid-upper lid full-thickness laceration wound, which involved the lid margin and extended 5 mm vertically. Additionally, a full-thickness crescent-shaped corneal laceration wound extended from seven o'clock (2 mm from limbus) up to 10 o'clock (4 mm from limbus) with vitreous prolapse externally. The anterior chamber (AC) was filled with hyphema. Right eye upper lids suturing, corneal laceration primary suturing, and dry anterior vitrectomy were performed.

On the other hand, the left eye findings revealed a vertically linear corneoscleral laceration wound at six o'clock, measuring 11 mm (scleral wound of 7.5 mm and corneal wound of 3.5 mm). There were uveal tissues mixed with vitreous content prolapsed out from the wound and hazy AC with hyphema. Left eye corneoscleral laceration repair and dry anterior vitrectomy were done. As the prolapsed uveal tissue was not viable, it was excised and removed. At the end of the surgery, both eyes were given intravitreal vancomycin (2 mg in 0.1 ml) and ceftazidime (2 mg in 0.1 ml) as endophthalmitis prophylaxis.

Postoperatively, the patient was started on systemic ciprofloxacin for two weeks with topical moxifloxacin and dexamethasone. Her visual acuity remained LP. Both eyeballs were firm with an intraocular pressure (IOP) of 26 mmHg. Topical timolol and brimonidine were started. Ultrasound B-scan showed bilateral retinal detachment with vitreous hemorrhage. The patient was informed of the poor visual prognosis, and she declined further intervention at that point in time.

**Figure 3:**
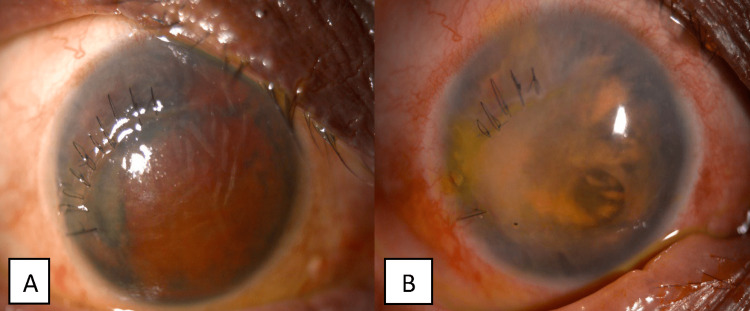
Right eye anterior segment photo on follow-up. (A) First month. The cornea was edematous. The anterior chamber (AC) was filled with blood clots and macerated lens matter. (B) Third month. A contracted old brownish blood clot was seen in the AC.

**Figure 4:**
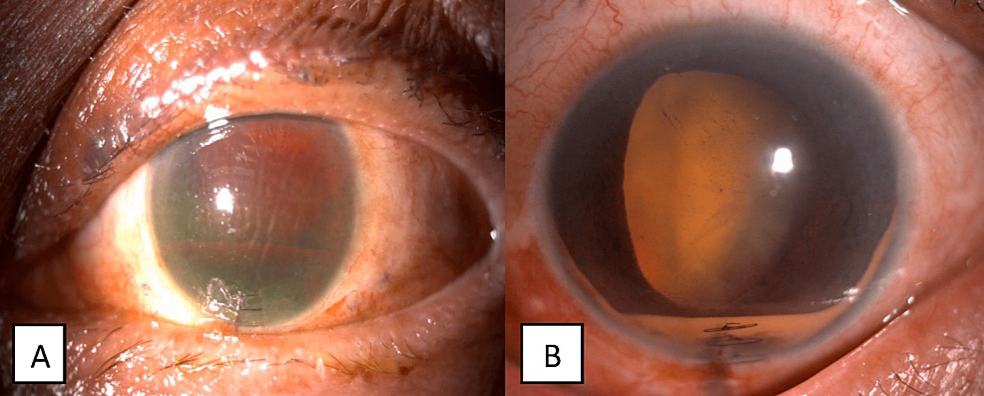
Left eye anterior segment photo on follow-up. (A) First month. The cornea was edematous with grade I hyphema. (B) Third month. Presence of old hyphema level inferiorly and cataractous lens.

During subsequent follow-ups in the first and third months, her visual acuity remained LP bilaterally. The AC for the right eye was deep, with almost total aniridia, and macerated lens matter seen and the IOP was 4 mmHg. Her left eye showed the AC was deep with cells 4+, old hyphema level inferiorly, and cataractous lens with an IOP 8 mmHg (Figures 3, 4). There was no sign of endophthalmitis. She defaulted her follow-up since then.

## Discussion

The ocular injury caused by durian varies from trivial non-sight-threatening injuries to sight-threatening injuries. So far, there were only five cases of ocular durian injury reported based on our literature review [3-6]. All of them sustained only uniocular injury from durian fall. Unfortunately, our patient sustained a binocular injury with poor vision outcome. A literature search showed that the predictors for poor visual outcome in patients with OGIs included mechanism or type of injury, poor preoperative visual acuity, time lag between injury and surgery, presence of RAPD, size and location of the wound, presence of hyphema, uveal prolapse, vitreous prolapse, retinal detachment, and vitreous hemorrhage [7-9]. Most of these factors were found in our patient.

In ocular trauma cases, a CT scan is helpful in detecting any orbital wall fracture, intraocular foreign body, or globe rupture. The role of conventional plain film X-ray should not be ignored as it is also useful in detecting intraocular foreign bodies. However, an ultrasound B-scan might worsen the expelled intraocular content; hence it is not advisable to be done prior to primary repair surgery.

All ocular durian injury cases that were reported underwent surgical intervention. The same goes for other cases of OGIs in Malaysia [10,11]. Surgical exploration under anesthesia is warranted to determine the extent of the injury. The goals of primary corneal or scleral wound repair are to maintain a water-tight wound, prevent further damage to the intraocular tissue, and re-establish normal anatomical ocular relationships [9]. Topical, intravitreal, and systemic antibiotics are important as prophylaxis to prevent endophthalmitis. So far, no endophthalmitis case was reported following ocular durian injury.

Retinal detachment can occur immediately directly due to the trauma or later secondarily to traction of proliferative vitreoretinopathy. Repair of retinal detachment can be postponed until after the primary repair [12]. Indication for combined primary repair with vitrectomy is rare, except in cases where there is a concurrent organic intraocular foreign body, which carries a significant risk of endophthalmitis and retinal toxicity [13]. Factors that can lead to a more common two-stage approach include corneal involvement that obscures the posterior view, vitreous that is tightly adhered to the retina, the risk of expulsive hemorrhage, and the unavailability of a vitreoretinal surgeon in an emergency setting.

A primary enucleation or evisceration is not recommended unless the globe is not salvageable and primary repair is not possible [14]. In view of the risk of sympathetic ophthalmia, patients with OGIs need long follow up.

## Conclusions

Traumatic globe injury due to durian is uncommon. Prevention is the best approach; hence, one should be prudent to wear protective gear while in a durian orchard to avoid an unprecedented accident. Employers should regularly monitor safety precautions in hazardous areas to minimize the mortality and morbidity associated with injuries caused by durian fruit.
